# Increased physical activity, higher educational attainment, and the use of mobility aid are associated with self-esteem in people with physical disabilities

**DOI:** 10.3389/fpsyg.2023.1072709

**Published:** 2023-02-23

**Authors:** Majed M. Alhumaid, Mohamed Ahmed Said

**Affiliations:** ^1^Department of Physical Education, College of Education, King Faisal University, Al-Ahsa, Saudi Arabia; ^2^Higher Institute of Sport and Physical Education of Kef, University of Jandouba, Jendouba, Tunisia

**Keywords:** quality of life, educational level, assistive devices, multiple sclerosis, cerebral palsy, physical activity

## Abstract

**Background:**

High self-esteem can help people with disabilities overcome barriers and improve their mental health and well-being. This study sought to examine self-esteem levels among Saudis with physical disabilities based on socio-economic factors. It also aimed to determine the minimum weekly duration of physical activity performed by participants and examine its effects, along with those of other socio-economic factors, on participants’ self-esteem.

**Methods:**

A participant sample (*N* = 582) consisting of Saudi individuals aged 33.78 ± 9.81 years with physical disabilities (males, *n* = 289; females, *n* = 293) was recruited to participate in this study. Levels of self-esteem were measured using the Arabic version of the Rosenberg Self-Esteem Scale.

**Results:**

Compared to women, men demonstrated significantly higher levels of overall self-esteem, positive feelings, and negative feelings (*p* < 0.01). The respondents’ average levels of overall self-esteem (*p* < 0.001), positive feelings (*p* < 0.01), and negative feelings (*p* < 0.001) also varied by type of physical disability. Wheelchair-using participants had the highest values for self-esteem and positive feelings; cane-using participants or those who did not use mobility aids had the lowest values. Weighted least squares regression showed that weekly physical activity was the factor that most affected self-esteem (*β* = 0.002), followed by education level (*β* = 0.115), then type of mobility device used (*β* = −0.07).

**Conclusion:**

Increased weekly physical activity, higher education levels, and the use of mobility aids were the factors likely to improve the self-esteem of Saudis with physical disabilities.

## Introduction

A disability is a condition or function that is considered to be significantly reduced from the usual norm for an individual or group. The term is used to refer to a person’s ability to function and includes physical, sensory, cognitive, and mental disorders, as well as mental illness and various types of chronic disease (Disabled World, 2022). The World Health Organization (2021) stated that people with disabilities face barriers, stigma, and discrimination when accessing health and related services and strategies. These barriers often prevent people with disabilities from fully and effectively participating in society on an equal basis with others (Howard et al., 2022).

Up-to-date statistics indicate that in Saudi Arabia, the overall prevalence of disability was around 7.1%, of which 3.9% are affected by physical disabilities. These rates are expected to increase due to the continued increase in health risk factors such as obesity, physical inactivity, road accidents, and chronic diseases (Zahra et al., 2022). People with physical disabilities are sometimes stigmatized by their families, excluded from social gatherings, and sometimes are not authorized to receive family visits. The family perspective is based on simple notions of disability including helplessness, lifelong dependence, home isolation, poor quality of life, etc. (Al-Jadid, 2013).

High self-esteem (SE) can help people with disabilities overcome these barriers, simplify their daily lives, and improve their mental health and well-being. SE is usually defined as “the extent to which one prizes, values, approves, or likes oneself” or “the overall affective evaluation of one’s own worth, value, or importance” (Blascovich and Tomaka, 1991, p. 115). The benefits of high SE fall into two categories: increased initiative and pleasant feelings. People with high SE report that they are more likable, more attractive, have better relationships, and make a better impression on others than people with low SE. Conversely, low SE is associated with anxiety, depression, suicide, alcoholism, aggression, antisocial behaviors, and criminality (Serafin et al., 2022).

Self-esteem in people with disabilities can be mediated and moderated by several factors, including the occurrence of SE (Ekeland et al., 2005), perceived stigma and social relationships (Zhang et al., 2014), socioeconomic status (Twenge and Campbell, 2002), and support from family members or others (Stark et al., 2017). In a literature review on gender and age differences in SE, Bleidorn et al. (2016) reported that men generally had significantly higher levels of SE than women. However, despite the huge gender differences in SE, men and women often follow similar life trajectories. For both sexes, SE is relatively high during childhood, declines during adolescence, increases gradually throughout adulthood, and then declines in old age (Trzesniewski et al., 2013). Thus, we propose the following hypothesis:

*H1a*: Saudis with physical disabilities generally report lower levels of SE and men report significantly higher levels of SE than women.

*H1b*: The level of SE of people with physical disabilities depends on their age.

Relevant studies have also shown that physical activity (PA) has many positive effects on the SE of people with disabilities as mobility and exercise improve a person’s fitness and performance (Nemček, 2017). However, the extant literature indicates poor adherence to the prescription of PA among those with disabilities (Maynou et al., 2021). Liu et al.’s (2015) systematic review and meta-analysis identified that PA-based interventions were associated with significant improvements in overall personal achievement, self-concept, and SE. The authors also revealed that PA-based interventions are potentially important in mediating the effect of PA on SE and that such interventions produce more beneficial outcomes in school and gymnasium settings compared to other contexts. Ahn et al. (2021) also revealed that participation in sports activities increases the SE of individuals with disabilities, may help them overcome feelings of pain or sadness, and can positively influence their acceptance of their disability. Jung et al. (2022), in a four-year follow-up study assessing the impact of disability acceptance on SE in adults with disabilities, reported that uncompromising people with disabilities had reduced disability acceptance; moreover, when self-denial was maximized, there was a pathological risk of depression and suicide. It can also be accompanied by the social risks of selfish behavior and aggression. Therefore, it is necessary to care for people with disabilities to ensure that they have high SE by healthily accepting their disability, not only individually but also socially. Thus, we propose the following hypothesis:

*H2a*: A greater amount of weekly PA is associated with a higher level of SE in participants with physical disabilities.

The literature also indicates that there is a significant and mostly positive relationship between SE and education level (Zhao et al., 2021; Al Awaji et al., 2022). However, Vialle et al.’s (2005) study on the relationship between SE and the achievement of gifted students is at odds with this assertion: It reported that there is no significant difference in SE between gifted and non-gifted students. Besides, the study found no relationship between SE and academic performance in the gifted group. Studies have also shown that SE is strongly linked to opportunities to make meaningful decisions (Nemček et al., 2014; Sandjojo et al., 2019). A greater level of independence has been related to increased feelings of happiness and satisfaction and a higher quality of life (Sandjojo et al., 2019). An independent person is usually someone who has high levels of SE and self-worth. Independence is essential in life and plays a substantial role in what a person achieves in school, work, and relationships. This leads to a sense of accomplishment that will improve how they view themselves. Le Breton (2004) argues that risky behaviors may have benefits because they are associated with the development of autonomy and survival without the benefit of parental protection. It can also be argued that risky behaviors can promote autonomy in adolescence and are believed to help in forming an identity. Thus, we propose the following hypothesis:

*H2b*: Higher education level positively modulates SE among Saudis with physical disabilities.

Assistive devices are also widely regarded as powerful tools for increasing the independence of people with disabilities while simultaneously allowing them to participate in PAs and all tasks of daily living (Brown et al., 2011). By definition, assistive devices refer to products whose primary purpose is to support the functioning and independence of individuals with disabilities to promote their academic, social, and physical well-being (McNicholl et al., 2021). According to these authors, inadequate assistive device training, the inadequacy of assistive devices, the availability of external support, and the challenge of negotiating multiple sources of information can impede the effective use of assistive devices and thus limit individuals’ engagement in the higher education environment. Therefore, choosing the right assistive device is the key to effective assistance in all of these activities. Tamakloe and Agbenyega (2017) argued that assistive devices if used effectively, could create a positive environment for the independence and development of people with disabilities. Assistive devices allow people with disabilities to overcome their weaknesses by increasing the strength needed to reach their potential and improving their motivation (Robitaille, 2010). Although several studies indicate that technical assistance can improve the quality of life of people with disabilities, much less is said about the potential of everyday technical assistance to promote SE for these individuals (Nemček, 2021; Howard et al., 2022). This is a major omission, as assistive devices enable people to be more independent and engaged in PA through a wide range of participation, from observation to practice (Bryant and Bryant, 2011). Referring to the studies described above, we expect the following hypotheses:

*H2c*: Mobility assistance correlates with SE in people with physical disabilities.

*H2d*: The type of physical disability affects SE in people with physical disabilities.

This study sought to examine SE levels among Saudis with physical disabilities based on socio-economic factors such as gender, age, education level, type of physical disability, and the use of mobility aids. It also aimed to determine the minimum weekly duration of PA performed by participants and examine its effects, along with those of other socio-economic factors, on participants’ SE.

## Materials and methods

### Participants and procedure

The Arabic version of the Rosenberg Self-Esteem Scale (RSES) (Rosenberg, 1965) was used to examine the SE of people with physical disabilities in Saudi Arabia. This version was previously translated from English by ALAhmari et al. (2019), and our research group studied its validity and reliability in a group of volunteer participants with disabilities (*N* = 30). The construct validity was assessed using exploratory factor analysis and principal component analysis, while the reliability was assessed by Cronbach’s alpha. An electronic copy was then distributed through emails sent to representatives of the Association of Motor Disabled for Adult Mobility, located in the city of Riyadh, which caters to all Saudi people with physical disabilities. Some participants were also invited to participate in our study. Each email contained a Google Form link that led to a page with details about the study’s objectives and instructions for participants. All participants voluntarily participated in the study, and informed consent was obtained by asking participants to click on the questionnaire to begin. Participants were informed that the questionnaire would take approximately 10 min to complete. The results were then uploaded (*N* = 645) and checked for accuracy, and incomplete or disputed questionnaires were discarded (*n* = 63). In total, the results from 582 participants (289 males and 293 females) aged 18–59 years were stored and analyzed. Ethical approval was granted by the Research Ethics Board of King Faisal University, Saudi Arabia (KFU-REC-2021-DEC-EA000307).

#### Reliability and validity of the Arabic version of the Rosenberg self-esteem scale

The analysis revealed two factors in the Arabic version of the RSES: (1) Factor 1, which included Items 1, 3, 4, 7, and 10, labeled as positive feelings, and (2) Factor 2, which included Items 2, 5, 6, and 9, labeled as negative feelings. The eighth item was removed because its load factor was <0.30, whereas the remaining nine items achieved an acceptable item-to-total correlation. The positive factor alone explained 27.70% of the total variance; however, combined with the second factor, it indicated a cumulative eigenvalue of approximately 53.5% of the total variance, indicating good validity of the scale. Cronbach’s alpha was then calculated separately for the two factors and combined. The respective values were 0.79, 0.77, and 0.83, which indicated good reliability of the Arabic version of the RSES. Therefore, the validity and traceability were met, and the Arabic RSES was a reliable and valid measure of the SE of people with physical disabilities.

### Measures

#### Sociodemographic factors and weekly physical activity

The demographic form was used to collect data on age, gender, educational level, type of physical disability, type of physical assistive device used, PA participation in units/week, and daily PA duration in minutes/unit. The last two items were measured on a 4-point Likert scale based on responses to two questions indicating whether or not the participant engaged in physical activity/exercise. The first question asked about the number of days per week, while the second question asked about the duration of PA in minutes per session. The responses were used to calculate the minimum weekly PA time in minutes by multiplying the lowest reported number of sessions performed per week by the lowest reported time in minutes per session (Strath et al., 2013). The scoring method used distinguished 4 levels of weekly physical practice by adding four times the product of the range divided by four to zero (<50 min/week, from 50 to <100 min/week, from 100 to <150 min/week, and ≥ 150 min/week). The Cronbach’s alpha value of this study was 0.661 indicating an acceptable level of reliability (Taber, 2018).

### Assessment of the level of self-esteem

The Arabic version of the RSES whose validity and reliability have been previously verified was used. RSES is a 10-point scale that measures overall SE by measuring positive and negative feelings about oneself. The scale is meant to be uni-dimensional and all questions are answered on a 4-point Likert-type scale ranging from “strongly agree” (4 points) to “strongly disagree” (1 point) for items 1, 3, 4, 7, and 10 and vice versa (4 points for “strongly disagree” and 1 point for “strongly agree”) for items 2, 5, 6, 8, and 9. The RSES results have been interpreted differently in previous studies (Ryszewska-Łabędzka et al., 2022). In our study, we used the following scoring: the sum of all answers divided by 10 is taken as the participant’s score differentiated into 4 levels by adding four times the product of the range divided by four to one: scores of 1.0–1.75 indicate low SE; scores of 1.76–2.50 indicate moderate SE; scores of 2.51–3.25 indicate high SE; and scores of 3.26–4.0 indicate very high SE. The 10 statements of the questionnaire are as follows: (1) In general, I am satisfied with myself; (2) Sometimes I think I’m no good at all; (3) I feel that I have several good qualities; (4) I can do things as well as most people; (5) I feel that I do not have much to be proud of; (6) I feel useless sometimes; (7) I feel like a person of value, and that I am at least on an equal footing with others; (8) I wish I had more respect for myself; (9) In general, I tend to feel like a failure; and (10) I have a positive attitude toward myself.

### Statistical analysis

The first objective of the research was addressed by calculating the mean and standard deviation of the components of the RSES based on socioeconomic factors. The normality of the data distribution was tested using the Kolmogorov–Smirnov test, which found nonnormal distributions for all dependent variables. Data were compared using the Mann–Whitney nonparametric *U* test and the Kruskal–Wallis test. The second objective of the research was analyzed using weighted least squares (WLS) regression to deal with inefficiency-based problems due to biased estimates and standard errors resulting from unequal variance observed (violation of homoscedasticity; Akari and Gündoğdu, 2013). The linear regression assumptions were examined by examining the scatterplots of the matrix, the normality of the residuals, the relationship between all the independent variables, and the variance inflation factor values. These were obtained by regression analysis with the SE score as the dependent variable. The residuals were normally distributed according to the results of the Kolmogorov–Smirnov test, and the highest variance inflation factor value was 1.266; therefore, multicollinearity was ruled out. Finally, the independent variables were not strongly correlated with each other; therefore, the linearity of the data was checked. Statistical analysis was performed with SPSS 26 (IBM, United States) using a significance level of *p* < 0.05.

## Results

### Demographic data

A total of 582 Saudi participants with physical disabilities completed the RSES questionnaire, and their data were stored and analyzed. There were 289 men (49.66%) and 293 women (50.34%) between the ages of 18 and 59 (33.78 ± 9.81 years), including 276 participants (47.42%) aged 18–31, 208 participants (35.74%) aged 32–45, and 98 participants (16.84%) aged 46–59. A total of 133 of the participants (22.85%) did not use mobility aids; however, 449 participants (77.15%) used mobility assistive devices, including 335 who used wheelchairs, 55 who used crutches, and 38 who used canes. Most of the participants (71.65%) reported having a secondary school diploma (36.08%) or university-level degree and above (35.57%), while the remainder had a primary (14.09%) or intermediate (14.26%) degree. Approximately 46% of the respondents also reported having types of physical disabilities other than those listed in the questionnaire (Others); however, polio was the most prevalent disability across the study population (23.02%), followed by cerebral palsy, spinal diseases, progressive muscular dystrophy, and multiple sclerosis, with prevalence values of 9.8, 9.28, 8.93, and 3.44%, respectively.

### Self-esteem levels among participants with physical disabilities

Table 1 illustrates the SE levels of participants with disabilities stratified by the category of each independent variable. The results showed that the participants reported moderate overall SE (2.997 ± 0.516) levels, with moderately high positive feelings and low negative feelings. The participants’ average SE levels on the positive feelings factor was 3.22 ± 0.57, while the mean SE level related to the negative feelings factor was 3.01 ± 0.72. Compared to female participants, male participants reported significantly higher levels of overall SE, positive feelings, and negative feelings (*p* < 0.01 for all). Regarding the participants’ levels of education, the data showed that those who reported the lowest level of education (primary school) also reported the lowest levels of SE, positive feelings, and negative feelings (*p* < 0.01 for the intermediate level; *p* < 0.001 for the rest). No significant difference was found among the other groups stratified by level of education. The respondents’ average levels of overall SE (*p* < 0.001), positive feelings (*p* < 0.01), and negative feelings (*p* < 0.001) also varied by type of physical disability. The lowest values were observed in participants with multiple sclerosis and, to a lesser extent, in those with cerebral palsy; however, the highest values were noted in participants with poliomyelitis (Table 1). Importantly, significant differences (*p* < 0.001) were observed when comparing the positive feelings factor and the total scale among participants stratified by the type of mobility aid used. Participants using wheelchairs had the highest values of SE and positive feelings, while those using canes or those who did not use mobility aids had the lowest values. The levels of negative feelings did not differ significantly among groups stratified by the type of mobility assistive device used.

**Table 1:** Exploring the overall self-esteem and positive and negative feelings scores for independent variables (*N* = 582).

				Positive feelings score	Z/H	*p*	Negative feelings score	Z/H	*p*	Self-esteem score	Z/H	*p*
**Age (years)^KW^**												
1	18–31		276	3.25 (0.58)	3.819	NS	2.96 (0.77)	3.577	NS	2.98 (0.55)	1.915	NS
2	32–45		208	3.15 (0.57)			3.04 (0.66)			2.98 (0.48)		
3	46–59		98	3.24 (0.54)			3.11 (0.66)			3.06 (0.49)		
**Gender ^MW^**												
Male			289	3.28 (0.57)	−2.615	0.009	3.11 (0.76)	−3.778	0.001	3.06 (0.51)	−3.097	0.002
Female			293	3.15 (0.57)			2.92 (0.66)			2.94 (0.51)		
**Educational level^KW^**												
1	Primary		82	2.96 (0.59) ♣,†††,♠♠♠	19.939	0.001	2.72 (0.63)♣♣,†††,♠♠♠	21.168	0.001	2.76 (0.50)♣,†††,♠♠♠	23.055	0.001
2	Intermediate		83	3.21 (0.44)			3.00 (0.56)			2.97 (0.36)		
3	Secondary		210	3.22 (0.63)			3.08 (0.79)			3.02 (0.56)		
4	University level and above		207	3.30 (0.52)			3.07 (0.70)			3.08 (0.50)		
**Type of physical disability^KW^**												
1	Cerebral palsy		57	3.05 (0.63)♦♦,¶	17.584	0.004	2.78 (0.69) ♣♣,♦♦♦,¶	40.801	0.001	2.78 (0.56)♣♣,♦♦♦,¶¶	35.527	0.001
2	Spinal disease		54	3.21 (0.57)			3.13 (0.58)			3.09 (0.50)		
3	Progressive muscular dystrophy		52	3.10 (0.60)			2.84 (0.73)			2.89 (0.49) ♣,♦♦		
4	Sclerosis multiplex		20	2.87 (0.55) ♣,¶¶			2.51 (0.39)♣♣♣,†,♦♦♦,¶¶			2.65 (0.31)♣♣,♦♦♦,¶¶		
5	Poliomyelitis		134	3.32 (0.49) ††,♠			3.25 (0.68)†††,¶¶			3.13 (0.45)¶		
6	Others		265	3.24 (0.58)			2.99 (0.74)			3.00 (0.53)		
**Mobility assistive device^KW^**												
1	With	Wheelchair	356	3.23 (0.58)	1.983	NS	3.13 (0.65)††,♠♠♠	24.014	0.001	3.05 (0.52)††,♠♠♠	17.769	0.001
2		Crutches	55	3.19 (0.50)			3.01 (0.83)♠			2.99 (0.55)		
3		Cane	38	3.15 (0.62)			2.74 (0.76)			2.86 (0.43)		
4	Without (independent)		133	3.18 (0.56)			2.80 (0.77)			2.89 (0.51)		

^MW^The Mann–Whitney *U* Test was used; ^KW^The Kruskal–Wallis Test was used. Z/H, *Z* score or H-Value. ♣ < 0.05, ♣♣ < 0.01, ♣♣♣ < 0.001 differs from subgroup 2; † < 0.05, †† < 0.01, ††† < 0.001 differs from subgroup 3; ♠ < 0.05, ♠♠ < 0.01, ♠♠♠ < 0.001 differs from subgroup 4; ♦ < 0.05, ♦♦ < 0.01, ♦♦♦ < 0.001 differs from subgroup 5; ¶ < 0.05, ¶¶ < 0.01, ¶¶¶ < 0.001 differs from subgroup 6.

### Physical activity levels according to sociodemographic variables

**Figure 1:**
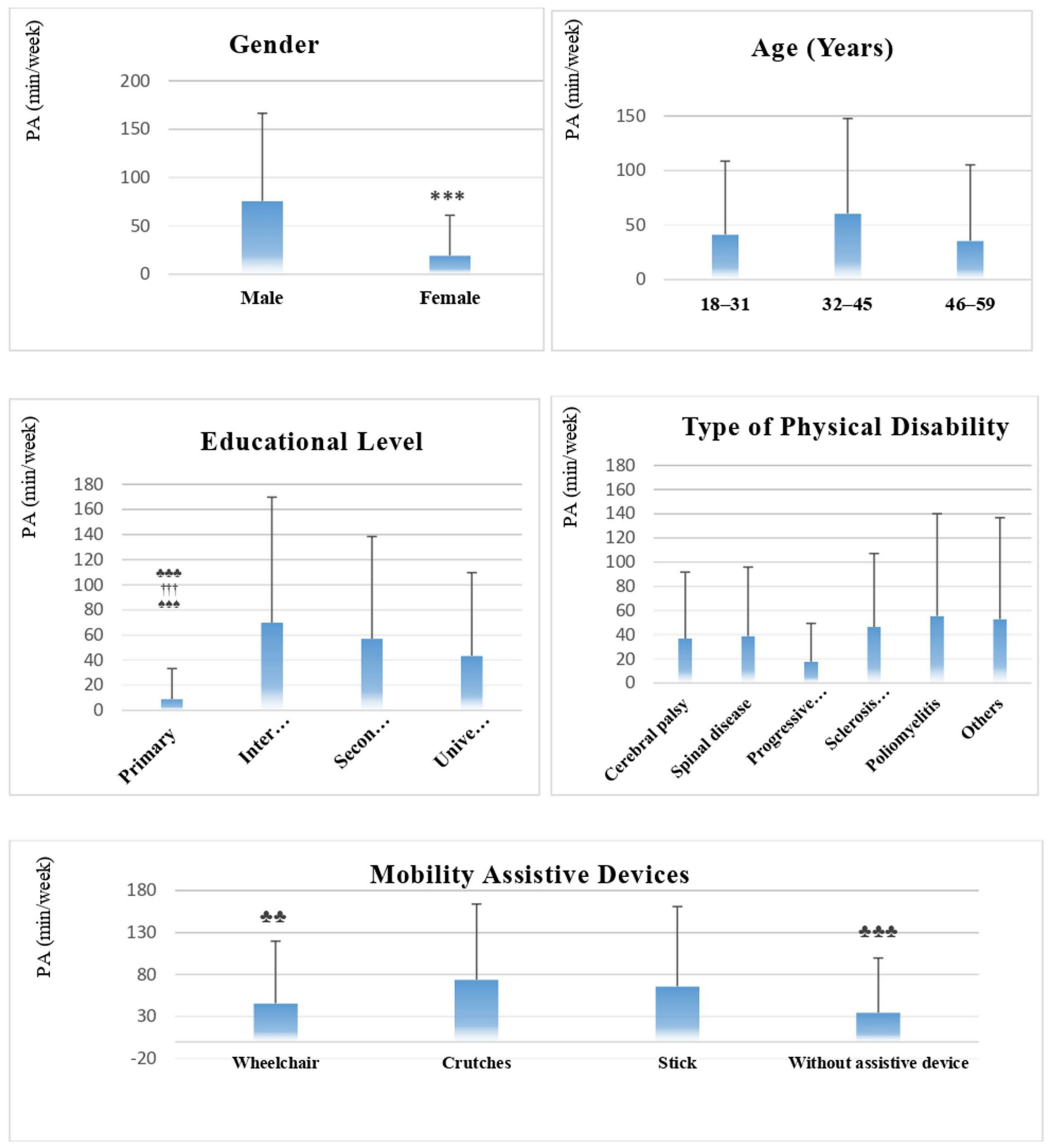
Weekly physical activity levels according to sociodemographic variables in Saudi participants with physical disabilities (*N* = 582). PA, physical activity. *** < 0.001 differs from subgroup 1; ♣♣ < 0.01, ♣♣♣ < 0.001 differs from subgroup 2; ††† < 0.001 differs from subgroup 3; ♠♠♠ < 0.001 differs from subgroup 4.

Figure 1 shows the total PA level and its subdomains. The level of PA in men was statistically higher than that in women (Kruskal–Wallis test, *p* < 0.001). A significantly shorter PA duration was also observed in the group of participants with a primary education level compared to the other three groups stratified by level of education (*p* < 0.001). No significant differences were noted between groups stratified by age category or by type of disability (Figure 1). However, multiple comparisons showed that participants using crutches as physical assistive devices were significantly more active than those using wheelchairs (*p* < 0.01) and those who did not use mobility aids (*p* < 0.001).

### Self-esteem at different levels of physical activity

**Figure 2:**
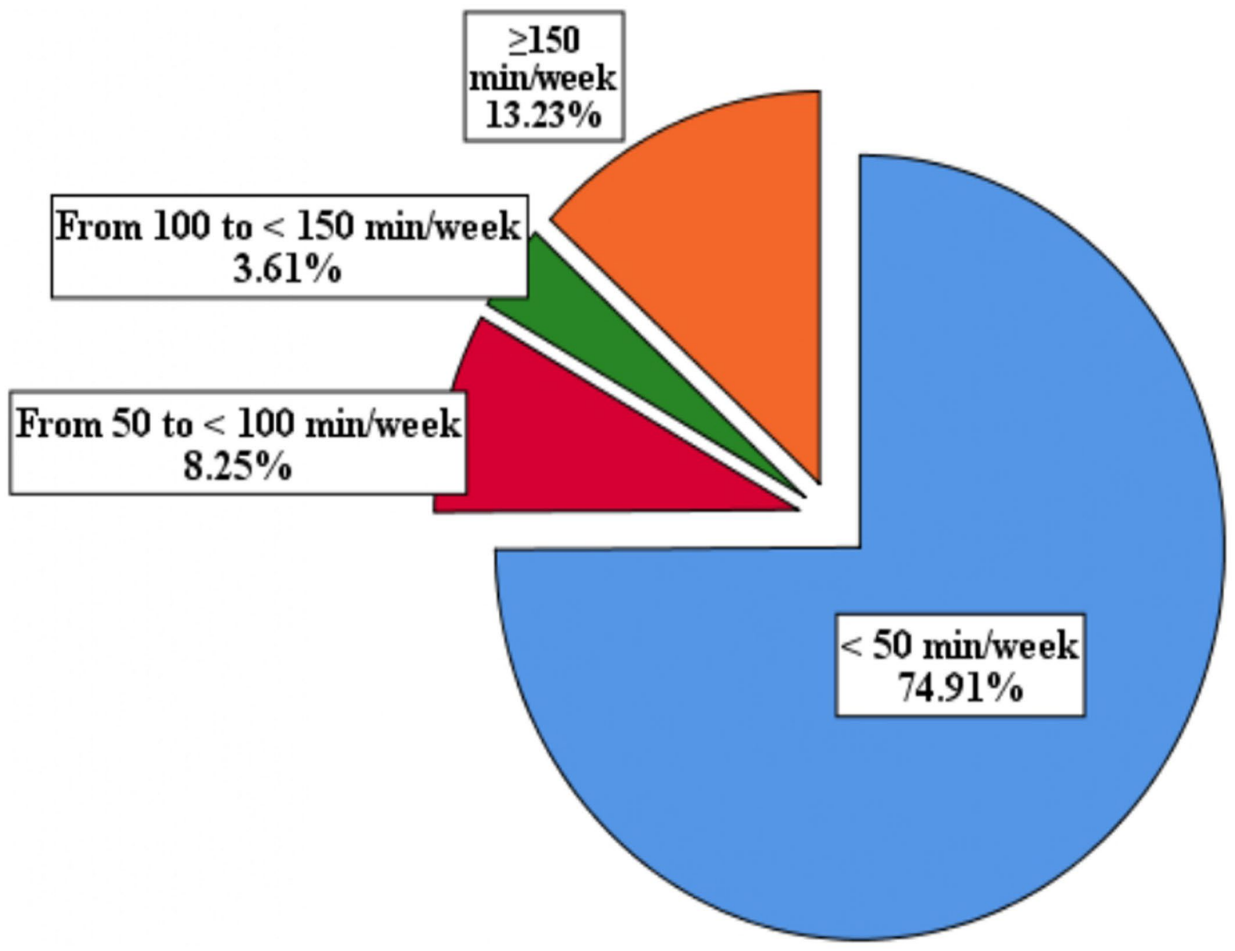
Exploration of weekly physical activity duration in Saudi participants with physical disabilities (*N* = 582).

**Figure 3:**
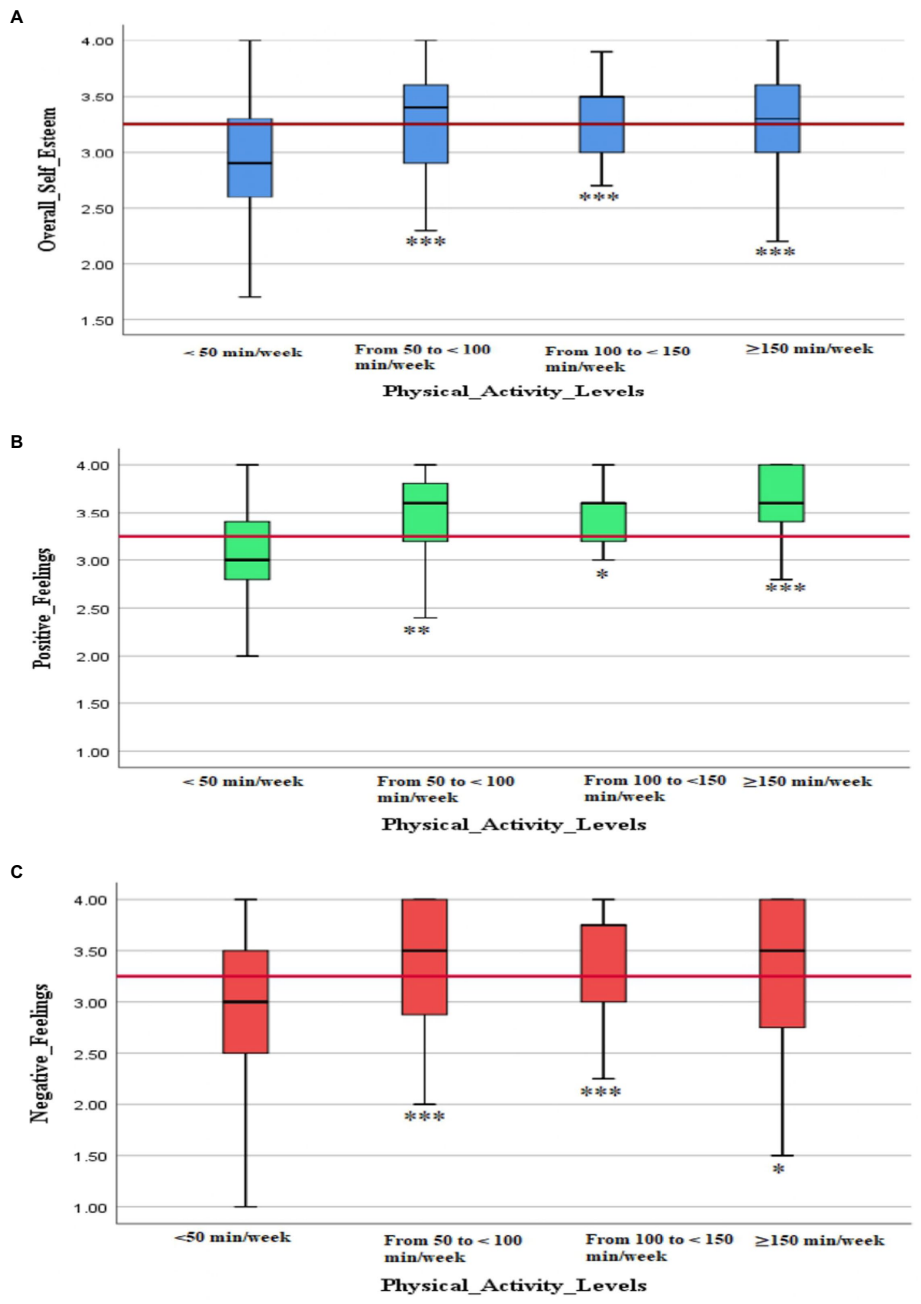
Levels of overall self-esteem **(A)** and positive **(B)** and negative **(C)** feelings according to the weekly physical activity level in Saudi participants with physical disabilities (*N* = 582). * < 0.05, ** < 0.01, *** < 0.001 differs from subgroup 1.

According to the World Health Organization’s (2020) PA recommendations for adults with disabilities, only 13.23% of the surveyed population achieved the required weekly amount of PA (150–300 min/week; Figure 2). However, 74.91% of the participants reported practicing PA for less than 50 min/week, 8.25% reported practicing PA for 50 to <100 min/week, and 3.61% reported practicing PA for 100 to <150 min/week. The data also showed that participants who were physically active for less than 50 min/week had the lowest levels of SE and positive and negative feelings. No significant differences were observed among the other groups (Figures 3A–C).

### Predictors of self-esteem

Weighted least squares regression showed that a significant regression pattern was identified among participants with physical disabilities, with *r*-squared values of 0.138 for positive feelings, 0.143 for negative feelings, and 0.161 for overall SE (Table 2). Therefore, the regression analysis results indicated that the independent variables accounted for 13.8, 14.3, and 16.1% of the variation in the SE levels of the participants for the positive feelings factor, negative feelings factor, and the total scale, respectively. Variance inflation factor values varied between 1.041 and 1.33 for all predictors, indicating a lack of collinearity in the results and therefore sufficient statistical significance. Of note, age, gender, and disability type were not labeled as potential covariates and were considered regressors of overall SE, positive feelings, and negative feelings. This is due to the lack of correlation (or weak correlation) observed between these variables and the dependent variables, and the negligible effects of these parameters on the regression results. Indeed, the WLS was calculated four consecutive times for each dependent variable and each time gender, age, or disability type was subtracted with no noticeable change in the association of the other regressors with the dependent variables (Supplementary Material).

**Table 2:** Weighted least squares regression models for the effect of sociodemographic factors and weekly physical activity on the levels of overall self-esteem and positive and negative feelings among Saudi participants with physical disabilities (*N* = 582).

		Unstandardized coefficients		*R*^2^	*t*	Sig.	95.0% CI for *β*		VIF
		*β*	Std. error				Lower bound	Upper bound	
Overall self-esteem	(Constant)	2.498	0.144	0.161	17.328	0.001	2.215	2.781	
	Gender	0.011	0.045		0.256	NS	−0.076	0.099	1.306
	Age (years)	0.002	0.002		0.978	NS	−0.002	0.006	1.159
	Type of physical disability	0.029	0.012		2.469	0.014	0.006	0.052	1.041
	Educational level	0.115	0.019		5.908	0.001	0.077	0.153	1.096
	Mobility assistive device	−0.07	0.016		−4.39	0.001	−0.101	−0.038	1.111
	Weekly Physical Activity	0.002	0.000		6.162	0.001	0.001	0.002	1.213
Positive feelings	(Constant)	2.883	0.163	0.138	17.671	0.001	2.563	3.204	
	Gender	−0.015	0.051		−0.306	NS	−0.115	0.084	1.33
	Age (years)	−0.003	0.002		−1.232	NS	−0.008	0.002	1.141
	Type of physical disability	0.029	0.013		2.196	0.028	0.003	0.055	1.052
	Educational level	0.101	0.023		4.429	0.001	0.056	0.146	1.089
	Mobility assistive device	−0.034	0.018		−1.889	NS	−0.07	0.001	1.122
	Weekly physical activity	0.002	0.000		6.493	0.001	0.001	0.002	1.245
Negative feelings	(Constant)	2.442	0.196	0.143	12.455	0.001	2.057	2.827	
	Gender	0.060	0.062		0.967	NS	−0.061	0.181	1.289
	Age (years)	0.004	0.003		1.47	NS	−0.001	0.01	1.15
	Type of physical disability	0.033	0.015		2.124	0.034	0.002	0.063	1.049
	Educational level	0.133	0.026		5.167	0.001	0.082	0.183	1.107
	Mobility assistive device	−0.153	0.024		−6.517	0.001	−0.2	−0.107	1.094
	Weekly physical activity	0.002	0.000		4.753	0.001	0.001	0.003	1.235

Age, gender, and disability type were considered as regressors. NS, not significant; VIF, variance inflation factor.

Table 2 shows the extent, direction, and strength of the relationships between individual predictors and levels of overall SE, positive feelings, and negative feelings in the overall sample. Note that the scores for the items related to negative feelings were reversed during data processing. Weekly PA, as the first predictor of positive beta scores, suggested that participants with physical disabilities could increase their levels of SE and its two related subdomains (positive feelings factor and negative feelings factor) by increasing their amount of weekly PA. The *β* values were 0.002 for all groups. Educational level (*β* = 0.115; *p* < 0.001), as the second predictor that achieved positive beta scores, suggested that participants with higher levels of education had higher levels for SE and its subdomains. The type of physical disability was the third factor that positively impacted SE and the magnitude of positive and negative feelings, suggesting that participants with polio had the highest SE, while those with sclerosis multiplex and cerebral palsy had the lowest. The values of *β* were 0.029, 0.033, and 0.029, respectively. The type of mobility aid used also had an impact on SE but returned only negative feelings. The *β* scores were −0.07 and −0.153, respectively, indicating that participants who used wheelchairs had the highest levels of SE and negative feelings; however, those who used canes or did not use mobility aids had the lowest scores.

## Discussion

The purpose of this study was to examine the levels of SE among Saudis with physical disabilities and, at the same time, to select and highlight the factors that most enhance positive feelings and overall SE and reduce negative feelings. The results showed that Saudis with physical disabilities reported moderate overall SE, with significant inferiority among women compared to men and among participants with primary education compared to those with higher levels of education. There was no effect of age on the participants’ SE. Extensive research has identified that gender and age have clear effects on the level of SE (Bleidorn et al., 2016). Regardless of cohort, sample, or measure, men tend to have higher SE than women, and regardless of gender, all individuals show an increase in SE from late adolescence to middle adulthood before it narrows in old age. The reported effect size is almost the same at all life stages, varying from low to moderate, except in adolescence where it tends to increase slightly. These two effects are now considered the most established findings in the SE literature (Trzesniewski et al., 2013; Orth and Robins, 2014). These studies also suggest that gender differences are determined, at least in part, by universal mechanisms that reflect both universal sociocultural factors and genetic biological processes that transcend culture and context (Gao et al., 2020; Liu, 2022). Moderate global SE, which was slightly higher than that in our study [2.997 (0.516) vs. 3.14 (0.56)], was also reported in 292 Saudi people with disabilities, with a clear male superiority over females. Narimani and Mousazadeh (2010) also reported lower SE scores in people with physical disabilities than in those without disabilities and in men than in women. Heydari et al. (2009), examining SE levels between students with and without disabilities, found that SE and life satisfaction were lower in people with physical disabilities than in those without physical disabilities. Nemček (2016) also confirmed lower SE in a group of sedentary people with disabilities compared to a comparable group of healthy people. However, contrary to our results, this author surveyed people with different types of disabilities and found no significant gender differences in SE scores, but the mean scores showed higher SE in females than in males (Nemček, 2013).

The lower levels of SE in people with disabilities can be explained by noting that body limb defects and injuries are important determining factors in the structure of human personality, so scientists know that self-depreciation is a consequence of personality defects and disorders. Therefore, based on Adler’s psychological principles, any factor that robs a person of their pride and SE can be a major factor causing senseless emotions. Such a factor can turn a person into a mentally ill and insane person. These results show that SE is lower in people with disabilities (Narimani and Mousazadeh, 2010). Regarding gender differences, Twenge and Campbell (2002) suggested that one reason for the differences between men and women is that women’s participation in forced labor has increased significantly over time. While women have increasingly pursued professional careers in recent decades, the changes for men during this time have been more subtle. Men’s participation in the labor force has not changed, so they no longer dominate occupations as they used to. In addition, men have given up the role of breadwinner, as fewer and fewer men are solely responsible for supporting their families. These shifts in gender roles have increased the psychological centrality of socioeconomic status to women’s SE and decreased it for men’s SE. It is important to note that only beginning in 2017 were Saudi women allowed to drive vehicles and access government services such as education and health care without requiring the consent of a male guardian. This may partly explain the lower SE of Saudi women compared to men, but this is likely to change in the future.

The Social Model of Disability (Oliver, 1983) suggests that to improve the quality of life of people with disabilities, we must first change societal attitudes (Hughes and Paterson, 2006). This includes removing stigma and reducing social inequalities while empowering people with disabilities. In this sense, PA was considered an important factor that can improve SE in people with disabilities, although research shows that the participation rates of people with physical disabilities remain lower than those of older comparison groups without disabilities. Hollis et al. (2020) found that less than half (45.2%) of US adults with motor disabilities engaged in aerobic PA, and 39.5% met one or both PA guidelines. The same data collected from Americans in 1997 revealed that compared to 16% of people without disabilities, only 12% of people with disabilities engaged in moderate PA for at least 30 min a day, 5 days a week. If only leisure-time PA was considered, the difference between people with and without disabilities was even greater: 56 and 36%, respectively, engaged in no leisure-time PA (U.S. Department of Health and Human Services, 2000). Alfermann and Stoll (2000) reported a significant association between PA and higher levels of SE in healthy middle-aged adults. In a longitudinal cohort study among kindergarten to fourth-grade females, Noordstar et al. (2016) found that changes in global SE were significantly associated with perceived athletic proficiency and moderate-to-vigorous PA. Recently, Romero et al. (2022) reported on the positive effects of endurance and strength exercises on SE in male college students. There was a statistically significant relationship between exercise frequency and SE, while the relationship between exercise type and SE was not significant.

Nevertheless, the association between PA and SE in people with physical disabilities remains unclear (Jalayondeja et al., 2016). Crawford et al. (2008) found that high fitness levels in people with physical disabilities affect the likelihood of returning to as-normal-as-possible community life. These authors also noted that highly active people with physical disabilities participated more in recreational and social activities than inactive non-disabled people. In contrast, Gutierrez et al. (2007) examined the association between subjective quality of life, PA, and community involvement among 80 paraplegic subjects with shoulder pain, and found a weak correlation between PA, quality of life, and community involvement. Also, Jalayondeja et al. (2016) revealed that while PA did not significantly explain the quality of life, 34.7% of the quality of life was explained by SE. These authors also found that those with disabilities who reported good quality of life engaged in high-intensity PA compared to those who reported a fair to poor quality of life.

Importantly, the results of the present study showed that among Saudi Arabians with physical disabilities, there was a positive association between participation in PA and SE. Our results showed that at least 40 min/week of PA resulted in significant improvements in the levels of positive feelings and overall SE and, to a lesser extent, in the level of negative feelings. Moreover, and in accordance with the practical recommendations required by different health organizations, a minimum of 150 min/week of PA provides more benefits for SE and its components. Participation in PA appears to be a critical predictor of SE. In other words, people with physical disabilities who are physically active tend to report higher levels of SE than those who are inactive. This finding largely supports the work of other studies on different types of disabilities reaching a consensus that exercise can effectively improve the SE of people with disabilities (Yan et al., 2019; Shang et al., 2021). Nemček et al. (2014) found that the differences between active and sedentary people with disabilities show that those who preferred active lifestyles and played sports (elite and all levels of sports) were happier with their lives than those who did not exercise. Therefore, it has been recognized that to promote these benefits for people with disabilities, work must be directed toward increasing opportunities for their participation in PA and sports (Groff et al., 2009). This is particularly important given the societal shift from segregation to inclusion for people with physical disabilities and can therefore be further facilitated through PA and sports. It is believed that people with disabilities can also benefit from physically active lifestyles. People’s health states and secondary health problems can lead to problems in everyday life (disability). Such functional problems and, in particular, mobility problems can be positively influenced by a physically active lifestyle and thus reduce disabilities. In addition, secondary health and functioning problems in people with disabilities that could be prevented or reduced by a physically active lifestyle include the risk of coronary artery disease, type 2 diabetes mellitus, osteoporosis, osteoarthritis, colon cancer, high blood pressure, decreased balance, decreased health-related fitness, spasticity, weight problems including obesity, depression, urinary tract infections, decreased SE, impaired ability to have normal social interactions and increased dependence on others. This shows that a physically active lifestyle is probably even more important for the health and well-being of people with disabilities than for the general population (van der Ploeg and Bull, 2020). In contrast, other studies have shown much smaller and even non-significant associations between PA and SE among people with disabilities. According to Bondár et al. (2020), these inconsistent findings likely reflect the methodological differences between the studies mainly in terms of the conceptual measures of SE and the prescribed activity types, intensities, and durations of PA.

The literature also supports our claim that SE is associated with the level of education. Bano et al. (2015) compared the levels of SE in students with and without disabilities and found that both groups of participants had high levels of SE. The result also showed that disability and gender did not significantly affect the students’ levels of SE. The study concluded that education plays an important role in improving students’ SE and eliminating gender-related stigma. The study also concluded that the provision of education can lead students with disabilities to recognize, accept and use their skills. This will also increase their value in their ranking (Bano et al., 2015). Previous studies have also shown that higher levels of education lead to a better quality of life, especially regarding psychology and the environment (Jani et al., 2020). Naturally, a higher education level is likely to help people with disabilities understand their rights, gain a higher level of respect, find better jobs, and improve their interpersonal relationships (Singal et al., 2015). In general, education improves SE and well-being by providing access to non-alienated economic and labor resources; this increases feelings of control over life, as well as access to stable social relationships such as marriage, which increases social support (Addabbo et al., 2016). In contrast, Jalayondeja et al.’s (2016) study of the associations between quality of life, education, PA, and SE found that quality of life was explained by SE and daily life, but was not significantly related to education level. The differences may be explained by the fact that all the participants were in vocational education and training at school. The authors also reported that all respondents who reported having a good quality of life claimed that they were satisfied with education and training that matched their skills and interests for their future careers.

The type of physical disability and the assistive device used can also affect SE scores. The lowest SE among our participants was found in patients with multiple sclerosis or cerebral palsy, while the highest was found in participants with polio. Participants who used wheelchairs also had higher SE and positive feelings than those who used other assistive devices. According to Miyahara and Piek (2011), people with physical disabilities may be concerned about their functional disabilities, body structure, and appearance, which may not conform to sociocultural norms. Therefore, a physical disability is considered an obstacle to development. The inconsistency of the impact of physical disabilities on people with disabilities could be explained by the multidimensional interactive model of self-concept, which takes multiple dimensions of the self and the socio-cultural impact on the individual into account. From this perspective, self-perceptions in several domains could be interpreted as reflecting, or at least being influenced by, the sociocultural values in each dimension. Jung et al. (2022) found that low disability acceptance contributed to low SE. On the Disability Acceptance Scale, adults who were struggling with their disability were more likely to have low SE. This may be because hiding the limitations of their disability made them more aware of their alienation. To overcome their sense of inferiority, they refused to accept their disability by distorting reality or fooling themselves, resulting in low SE. The greater the distortion process, the greater the disability and the greater the resulting integration difficulties. Disability paradox theory refers to people who are satisfied with themselves, can achieve their life goals, and enjoy a high quality of life despite or because of a disability. People who respond better to their disability have higher levels of SE, social participation, and quality of life (Brown et al., 2011). In other words, whether people with disabilities can hide their disability affects their disability acceptance and SE. Therefore, the acceptance of a disability requires that people avoid devaluing a person because of their disability, hiding the disability out of shame, and overestimating the disability. It is about recognizing the discomfort caused by the disability and trying to find and accept the reality and limitations that it brings (Jung et al., 2022).

The results of this study should be interpreted with some limitations in mind. First, although the research team made several efforts to minimize bias by making the Arabic version of the RSES clear and readable for all people, the team was unable to exclude responses or recall biases that may have affected the results. Second, regarding the different types of physical disabilities assessed, five types were identified (cerebral palsy, spinal diseases, progressive muscular dystrophy, multiple sclerosis, and poliomyelitis), while the rest were grouped under the heading “Other,” in which almost half of the participants were included. It is necessary to examine the impact of other specific disability types on SE. Focusing on other disability types may show implications beyond those found in our study for the types of disabilities already identified. Finally, the questionnaire was used as an indirect method to assess PA participation and duration, which does not exclude the possibility of recall bias and social desirability results. In addition, it was difficult to distinguish among the PA patterns the participants engaged in (aerobics, muscular strengthening, or a combination of aerobics and strengthening). Using direct measurement methods such as pedometers or motion sensors can provide much greater accuracy, mainly depending on the type and intensity of the PA performed (Hollis et al., 2020).

## Conclusion

The data showed that the participants reported moderate levels of overall SE. Compared to women, men demonstrated significantly higher levels of overall SE, positive feelings, and negative feelings. Participants with the lowest level of education (i.e., primary school) also reported the lowest levels of SE, positive feelings, and negative feelings. The respondents’ average levels of overall SE, positive feelings and negative feelings also varied depending on the type of physical disability and the type of mobility device used. WLS regression analysis noted that the factors influencing positive feelings included weekly PA, the level of education, and the type of physical disability; factors influencing negative feelings included weekly PA, the type of mobility assistance device, the level of education, and the type of physical disability; and factors influencing overall SE included weekly PA, the level of education, the type of mobility aid used, and the type of physical disability. More attention should be also given to participants with multiple sclerosis and cerebral palsy, and appropriate tools should be provided to all participants who need them.

The results of the present study may provide useful guidelines for the Saudi Arabian Ministry of Sport and other related organizations and associations for people with physical disabilities in terms of raising awareness of the importance of weekly PA for people with disabilities.

## Data availability statement

The raw data supporting the conclusions of this article will be made available by the authors, without undue reservation.

## Ethics statement

The studies involving human participants were reviewed and approved by Research Ethics Committee at King Faisal University, Saudi Arabia (protocol code KFU-REC-2021-DEC-EA000307, approved on 21 December 2021). The patients/participants provided their written informed consent to participate in this study.

## Author contributions

All authors listed have made a substantial, direct, and intellectual contribution to the work and approved it for publication.

## Funding

This research was funded by the Deanship of Scientific Research, King Faisal University, Saudi Arabia, grant number GRANT1276. The funder had no role in the design of the study; in the collection, analysis, or interpretation of data; in the writing of the manuscript, or in the decision to publish the results.

## Conflict of interest

The authors declare that the research was conducted in the absence of any commercial or financial relationships that could be construed as a potential conflict of interest.

The reviewer NH declared a shared affiliation with the author MS to the handling editor at the time of review.

## Publisher’s note

All claims expressed in this article are solely those of the authors and do not necessarily represent those of their affiliated organizations, or those of the publisher, the editors and the reviewers. Any product that may be evaluated in this article, or claim that may be made by its manufacturer, is not guaranteed or endorsed by the publisher.

## References

[ref1] Addabbo T. Sarti E. Sciulli D. (2016). Disability and life satisfaction in Italy. Appl. Res. Qual. Life 11, 925–954. doi: 10.1007/s11482-015-9412-0

[ref2] Ahn H. Lee K. So Y. (2021). The mediating effect of disability acceptance in individuals with spinal cord injury participating in sport for all. Int. J. Environ. Res. Public Health 18:10883. doi: 10.3390/ijerph182010883, PMID: 34682630 PMC8535784

[ref3] Akari M. Gündoğdu S. (2013). A comparison of weighted least square estimation and ordinary least square estimation for analysing weight-length relationship of Por’s goatfish (Upeneus pori ben-Tuvia & Golani, 1989) in Iskenderun Bay. J. Appl. Biol. Sci. 7, 46–50.

[ref4] Al Awaji N. Zaidi U. Awad S. S. Alroqaiba N. Aldhahi M. I. Alsaleh H.. (2022). Moderating effects of self-esteem on the relationship between communication anxiety and academic performance among female health college students during the COVID-19 pandemic. Int. J. Environ. Res. Public Health 19:13960. doi: 10.3390/ijerph192113960, PMID: 36360835 PMC9658700

[ref5] ALAhmari T. Alomar A. Z. ALBeeybe J. Asiri N. ALAjaji R. ALMasoud R.. (2019). Associations of self-esteem with body mass index and body image among Saudi college-age females. Eat. Weight Disord. 24, 1199–1207. doi: 10.1007/s40519-017-0471-0, PMID: 29282654

[ref6] Alfermann D. Stoll O. (2000). Effects of physical exercise on self-concept and well-being. Int. J. Sport Psychol. 31, 47–65.

[ref7] Al-Jadid M. S. (2013). Disability in Saudi Arabia. Saudi Med. J. 34, 453–460. 23677260

[ref8] Bano H. Anjum N. Pasha S. (2015). Differences in self-esteem of university students with and without disability. J. Educ. Res. 18, 114–124.

[ref9] Blascovich J. Tomaka J. (1991). “Measures of self-esteem” in Measures of Personality and Social Psychological Attitudes. eds. Robinson J. P. Shaver P. R. Wrightsman L. S. (New York: Academic Press), 115–160.

[ref10] Bleidorn W. Arslan R. C. Denissen J. J. A. Rentfrow P. J. Gebauer J. E. Potter J.. (2016). Age and gender differences in self-esteem—a cross-cultural window. J. Pers. Soc. Psychol. 111, 396–410. doi: 10.1037/pspp0000078, PMID: 26692356

[ref11] Bondár R. Z. di Fronso S. Bortoli L. Robazza C. Metsios G. S. Bertollo M. (2020). The effects of physical activity or sport-based interventions on psychological factors in adults with intellectual disabilities: a systematic review. J. Intellect. Disabil. Res. 64, 69–92. doi: 10.1111/jir.12699 31833138

[ref12] Brown D. J. McHugh D. Standen P. Evett L. Shopland N. Battersby S. (2011). Designing location-based learning experiences for people with intellectual disabilities and additional sensory impairments. Comput. Educ. 56, 11–20. doi: 10.1016/j.compedu.2010.04.014

[ref13] Bryant D. P. Bryant B. R. (2011). Assistive Technology for People With Disabilities. London: Pearson Higher Education.

[ref14] Crawford A. Hollingsworth H. H. Morgan K. Gray D. B. (2008). People with mobility impairments: physical activity and quality of participation. Disabil. Health J. 1, 7–13. doi: 10.1016/j.dhjo.2007.11.004 21122706

[ref15] Disabled World. (2022) Disabilities: Definition, types and models of disability. Disabled World. Available at: www.disabled-world.com/disability/types/ (Accessed July 11, 2022)

[ref16] Ekeland E. Heian F. Hagen K. B. (2005). Can exercise improve self-esteem in children and young people? A systematic review of randomized controlled trials. Br. J. Sports Med. 39, 792–798. doi: 10.1136/bjsm.2004.017707, PMID: 16244186 PMC1725055

[ref17] Gao W. Ping S. Liu X. (2020). Gender differences in depression, anxiety, and stress among college students: a longitudinal study from China. J. Affect. Disord. 263, 292–300. doi: 10.1016/j.jad.2019.11.121, PMID: 31818792

[ref18] Groff D. G. Lundberg N. R. Zabriskie R. B. (2009). Influence of adapted sport on quality of life: perceptions of athletes with cerebral palsy. Disabil. Rehabil. 31, 318–326. doi: 10.1080/09638280801976233, PMID: 18608427

[ref19] Gutierrez D. D. Thompson L. Kemp B. Mulroy S. J. Physical Therapy Clinical Research Network, & Rehabilitation Research and Training Center on Aging-Related Changes in Impairment for Persons Living with Physical Disabilities (2007). The relationship of shoulder pain intensity to quality of life, physical activity, and community participation in persons with paraplegia. J. Spinal Cord Med. 30, 251–255. doi: 10.1080/10790268.2007.11753933, PMID: 17684891 PMC2031955

[ref20] Heydari A. Mashak R. Darvishi H. (2009). Compare of the self-efficacy, loneliness, fear of success and satisfaction in physically disabled students with normal students in Ahvaz Islamic Azad University. New Find Psychol. 10, 7–26.

[ref21] Hollis N. D. Zhang Q. C. Cyrus A. C. Courtney-Long E. Watson K. Carroll D. D. (2020). Physical activity types among US adults with mobility disability, behavioral risk factor surveillance system, 2017. Disabil. Health J. 13:100888. doi: 10.1016/j.dhjo.2020.100888, PMID: 32061542 PMC7470910

[ref22] Howard J. Fisher Z. Kemp A. H. Lindsay S. Tasker L. H. Tree J. J. (2022). Exploring the barriers to using assistive technology for individuals with chronic conditions: a meta-synthesis review. Disabil. Rehabil. Assist. Technol. 17, 390–408. doi: 10.1080/17483107.2020.1788181, PMID: 32663110

[ref23] Hughes B. Paterson K. (2006). “The social model of disability and the disappearing body: towards a sociology of impairment” in Overcoming Disabling Barriers. ed. Barton L. (Oxford: Routledge), 101–117.

[ref24] Jalayondeja C. Jalyondeja W. Suttiwong J. Sullivan P. E. Nilanthi D. L. (2016). Physical activity, self-esteem, and quality of life among people with physical disability. Southeast Asian J. Trop. Med. Public Health 47, 546–558. 27405139

[ref25] Jani R. Alias A. A. Tumin M. (2020). Persons with disabilities’ education and quality of life: evidence from Malaysia. Int. J. Incl. Educ. 26, 753–765. doi: 10.1080/13603116.2020.1726511

[ref26] Jung Y. H. Kang S. H. Park E. C. Jang S. Y. (2022). Impact of the acceptance of disability on self-esteem among adults with disabilities: a four-year follow-up study. Int. J. Environ. Res. Public Health 19:3874. doi: 10.3390/ijerph19073874, PMID: 35409553 PMC8997373

[ref27] Le Breton D. (2004). Risk taking behaviours among young people. Bulletin de l’Academie Nationale de Medicine 188, 1313–1321. 15918660

[ref28] Liu C. (2022). Research on the influence of college students' participation in sports activities on their sense of inferiority based on self-esteem and general self-efficacy. Front. Psychol. 13:994209. doi: 10.3389/fpsyg.2022.994209, PMID: 36438383 PMC9686373

[ref29] Liu M. Wu L. Ming Q. (2015). How does physical activity intervention improve self-esteem and self-concept in children and adolescents? Evidence from a Meta-Analysis. PLoS One 10:e0134804. doi: 10.1371/journal.pone.0134804, PMID: 26241879 PMC4524727

[ref30] Maynou L. Hernández-Pizarro H. M. Errea Rodríguez M. (2021). The Association of Physical (in) activity with mental health. Differences between elder and younger populations: a systematic literature review. Int. J. Environ. Res. Public Health 18:4771. doi: 10.3390/ijerph18094771, PMID: 33947122 PMC8124550

[ref31] McNicholl A. Casey H. Desmond D. Gallagher P. (2021). The impact of assistive technology use for students with disabilities in higher education: a systematic review. Disabil. Rehabil. Assist. Technol. 16, 130–143. doi: 10.1080/17483107.2019.1642395, PMID: 31335220

[ref32] Miyahara M. Piek J. (2011). “Physical disabilities and self-esteem” in Encyclopedia of Adolescence. ed. Levesque R. J. R. (New York, NY: Springer).

[ref33] Narimani M. Mousazadeh T. (2010). Comparing self-esteem and self-concept of handicapped and normal students. Procedia Soc. Behav. Sci. 2, 1554–1557. doi: 10.1016/j.sbspro.2010.03.234

[ref34] Nemček D. (2013). “Life satisfaction of people with disabilities” in Theory and Practice in Adapted Physical Activity (Olsztyń, Poland: Olsztyńska szkola wyźsza Im. Józefa Rusieckiego), 46.

[ref35] Nemček D. (2016). Life satisfaction of people with disabilities: a comparison between active and sedentary individuals. Journal of Physical Education and Sport 16, 1084–1088. doi: 10.7752/jpes.2016.s2173

[ref36] Nemček D. (2017). Self-esteem in people with physical disabilities: differences between active and inactive individuals. Acta Fac. Educ. Phys. Univ. Comen. 57, 34–47. doi: 10.1515/afepuc-2017-0004

[ref37] Nemček D. (2021). Sport participation differences in the self-esteem of people with physical disabilities using assistive technology. AIP Conf. Proc. 2343:100005. doi: 10.1063/5.0047751

[ref38] Nemček D. Labudová J. Oršulová N. (2014). Self-esteem in people with disabilities. Acta Fac. Educ. Phys. Univ. Comen. 54, 33–42.

[ref39] Noordstar J. J. Van der Net J. Jak S. Helders P. J. Jongmans M. J. (2016). Global self-esteem, perceived athletic competence, and physical activity in children: a longitudinal cohort study. Psychol. Sport Exerc. 22, 83–90. doi: 10.1016/j.psychsport.2015.06.009

[ref40] Oliver M. (1983). Social Work with Disabled People (Practical Social Work). London: Macmillan.

[ref41] Orth U. Robins R. W. (2014). The development of self-esteem. Curr. Dir. Psychol. Sci. 23, 381–387. doi: 10.1177/0963721414547414

[ref42] Robitaille S. (2010). The Illustrated Guide to Assistive Technology and Devices. New York, NY: Demos Medical Publishing.

[ref43] Romero I. Kaye A. Poulin C. Petersen M. E. (2022). An analysis of the effects of frequency and type of physical activity on self-esteem in adolescent males. Georgetown Sci. Res. J. 2, 42–52. doi: 10.48091/gsr.v2i2.42

[ref44] Rosenberg M. (1965). Society and the Adolescent Self-Image. Princeton, NJ: Princeton University Press.

[ref45] Ryszewska-Łabędzka D. Tobis S. Kropińska S. Wieczorowska-Tobis K. Talarska D. (2022). The Association of Self-Esteem with the level of independent functioning and the primary demographic factors in persons over 60 years of age. Int. J. Environ. Res. Public Health 19:1996. doi: 10.3390/ijerph19041996, PMID: 35206185 PMC8871774

[ref46] Sandjojo J. Gebhardt W. A. Zedlitz A. M. E. E. Hoekman J. den Haan J. A. Evers A. W. M. (2019). Promoting Independence of people with intellectual disabilities: a focus group study perspectives from people with intellectual disabilities, legal representatives, and support staff. J. Policy Prac. Intellect. Disabil. 16, 37–52. doi: 10.1111/jppi.12265

[ref47] Serafin L. Strząska-Kliś Z. Kolbe G. Brzozowska P. Szwed I. Ostrowska A.. (2022). The relationship between perceived competence and self-esteem among novice nurses–a cross-sectional study. Ann. Med. 54, 484–494. doi: 10.1080/07853890.2022.2032820, PMID: 35132927 PMC8843132

[ref48] Shang Y. Xie H. D. Yang S. Y. (2021). The relationship between physical exercise and subjective well-being in college students: the mediating effect of body image and self-esteem. Front. Psychol. 12:658935. doi: 10.3389/fpsyg.2021.658935, PMID: 34122243 PMC8194825

[ref49] Singal N. Mahama Salifu E. Iddrisu K. Casely-Hayford L. Lundebye H. (2015). The impact of education in shaping lives: reflections of young people with disabilities in Ghana. Int. J. Incl. Educ. 19, 908–925. doi: 10.1080/13603116.2015.1018343

[ref50] Stark L. Asghar K. Meyer S. Yu G. Bakemore T. Poulton C.. (2017). The effect of gender norms on the association between violence and hope among girls in the Democratic Republic of the Congo. Global Mental Health 4:E1. doi: 10.1017/gmh.2016.31, PMID: 28596902 PMC5454793

[ref51] Strath S. J. Kaminsky L. A. Ainsworth B. E. Ekelund U. Freedson P. S. Gary R. A.. (2013). Guide to the assessment of physical activity: clinical and research applications: a scientific statement from the American Heart Association. Circulation 128, 2259–2279. doi: 10.1161/01.cir.0000435708.67487.da, PMID: 24126387

[ref52] Taber K. S. (2018). The use of Cronbach’s alpha when developing and reporting research instruments in science education. Res. Sci. Educ. 48, 1273–1296. doi: 10.1007/s11165-016-9602-2

[ref53] Tamakloe D. Agbenyega J. S. (2017). Exploring preschool teachers’ and support staff’s use and experiences of assistive technology with children with disabilities. Australas. J. Early Childhood 42, 29–36. doi: 10.23965/AJEC.42.2.04

[ref54] Trzesniewski K. Donnellan B. Robins R. W. (2013). “Development of self-esteem” in Self-Esteem. ed. Zeigler-Hill V. (New York, NY: Psychology Press), 60–79.

[ref55] Twenge J. M. Campbell W. K. (2002). Self-esteem and socioeconomic status: a meta-analytic review. Personal. Soc. Psychol. Rev. 6, 59–71. doi: 10.1207/S15327957PSPR0601_3

[ref56] U.S. Department of Health and Human Services. (2000). Healthy People 2010: Understanding and Improving Health. 2nd Edn. Washington, DC: U.S. Government Printing Office.

[ref57] van der Ploeg H. P. Bull F. C. (2020). Invest in physical activity to protect and promote health: the 2020 who guidelines on physical activity and sedentary behaviour. Int. J. Behav. Nutr. Phys. Act. 17:145. doi: 10.1186/s12966-020-01051-1, PMID: 33239047 PMC7688396

[ref58] Vialle W. J. Heaven P. C. L. Ciarrochi J. V. (2005). The relationship between self-esteem and academic achievement in high ability students: evidence from the Wollongong youth study. Australas. J. Gifted Educ. 14, 39–45. doi: 10.21505/ajge.2015.0013

[ref59] World Health Organization. (2020). Guidelines on physical activity and sedentary behaviour. Geneva: World Health Organization. Available at: https://apps.who.int/iris/bitstream/handle/10665/336656/9789240015128-eng.pdf?sequence=1&isAllowed=y (Accessed July 15, 2022)

[ref60] World Health Organization. (2021). Disability and health. Available at: https://www.who.int/health-topics (Accessed July 12, 2022)

[ref61] Yan J. Li Q. Zhang Z. K. Wang B. Y. Zhu F. S. (2019). The influence of extracurricular physical exercise on the physical self-esteem and self-confidence of elementary students. Sports Sci. 40, 100–104. doi: 10.13598/j.issn1004-4590.2019.02.012

[ref62] Zahra A. Hassan M. S. Park J. H. Hassan S. U. Parveen N. (2022). Role of environmental quality of life in physical activity status of individuals with and without physical disabilities in Saudi Arabia. Int. J. Environ. Res. Public Health 19:4228. doi: 10.3390/ijerph19074228, PMID: 35409909 PMC8998774

[ref63] Zhang L. Wentao L. Liu B. Xie W. (2014). Self-esteem as mediator and moderator of the relationship between stigma perception and social alienation of Chinese adults with disability. Disabil. Health J. 7, 119–123. doi: 10.1016/j.dhjo.2013.07.004, PMID: 24411516

[ref64] Zhao Y. Zheng Z. Pan C. Zhou L. (2021). Self-esteem and academic engagement among adolescents: a moderated mediation model. Front. Psychol. 12:690828. doi: 10.3389/fpsyg.2021.690828, PMID: 34149576 PMC8209251

