# Supplementary material for: Increased physical activity, higher educational attainment, and the use of mobility aid are associated with self-esteem in people with physical disabilities
Source: Front Psychol. 2023 Feb 23;14:1072709. doi: 10.3389/fpsyg.2023.1072709 (PMC9995828; doi:10.3389/fpsyg.2023.1072709)
Supplement: Supplementary file 1 [file Data_Sheet_1.docx]

**Overall self-esteem**

***1- All demographic variables***

| Model | | Unstandardized Coefficients | | Standardized Coefficients | t | Sig. | 95.0% Confidence Interval for B | | Collinearity Statistics | |
| --- | --- | --- | --- | --- | --- | --- | --- | --- | --- | --- |
|  |  | B | Std. Error | Beta |  |  | Lower Bound | Upper Bound | Tolerance | VIF |
| 1 | (Constant) | 2.498 | .144 |  | 17.328 | .000 | 2.215 | 2.781 |  |  |
|  | Gender | .011 | .045 | .011 | .256 | .798 | -.076 | .099 | .766 | 1.306 |
|  | Age (years) | .002 | .002 | .040 | .978 | .328 | -.002 | .006 | .863 | 1.159 |
|  | Type of physical disability | .029 | .012 | .096 | 2.469 | .014 | .006 | .052 | .961 | 1.041 |
|  | Mobility assistive device | -.070 | .016 | -.177 | -4.390 | .000 | -.101 | -.038 | .900 | 1.111 |
|  | Educational level | .115 | .019 | .236 | 5.908 | .000 | .077 | .153 | .912 | 1.096 |
|  | Weekly Physical Activity | .002 | .000 | .259 | 6.162 | .000 | .001 | .002 | .825 | 1.213 |
| a. Dependent Variable: RSES score | | | | | | | | | | |
| b. Weighted Least Squares Regression - Weighted by weight RSES score | | | | | | | | | | |

***2- Without age***

| Model | | Unstandardized Coefficients | | Standardized Coefficients | t | Sig. | 95.0% Confidence Interval for B | | Collinearity Statistics | |
| --- | --- | --- | --- | --- | --- | --- | --- | --- | --- | --- |
|  |  | B | Std. Error | Beta |  |  | Lower Bound | Upper Bound | Tolerance | VIF |
| 1 | (Constant) | 2.596 | .104 |  | 25.027 | .000 | 2.392 | 2.800 |  |  |
|  | Gender | .002 | .043 | .002 | .043 | .966 | -.084 | .087 | .804 | 1.243 |
|  | Type of physical disability | .030 | .012 | .100 | 2.585 | .010 | .007 | .053 | .971 | 1.030 |
|  | Mobility assistive device | -.072 | .016 | -.182 | -4.550 | .000 | -.102 | -.041 | .914 | 1.093 |
|  | Educational level | .110 | .019 | .227 | 5.839 | .000 | .073 | .148 | .963 | 1.039 |
|  | Weekly Physical Activity | .002 | .000 | .257 | 6.120 | .000 | .001 | .002 | .827 | 1.210 |
| a. Dependent Variable: RSES score | | | | | | | | | | |
| b. Weighted Least Squares Regression - Weighted by weight RSES score | | | | | | | | | | |

***3- Without gender***

| Model | | Unstandardized Coefficients | | Standardized Coefficients | t | Sig. | 95.0% Confidence Interval for B | | Collinearity Statistics | |
| --- | --- | --- | --- | --- | --- | --- | --- | --- | --- | --- |
|  |  | B | Std. Error | Beta |  |  | Lower Bound | Upper Bound | Tolerance | VIF |
| 1 | (Constant) | 2.520 | .116 |  | 21.803 | .000 | 2.293 | 2.747 |  |  |
|  | Age (years) | .002 | .002 | .038 | .946 | .345 | -.002 | .006 | .906 | 1.104 |
|  | Type of physical disability | .029 | .012 | .097 | 2.484 | .013 | .006 | .052 | .962 | 1.039 |
|  | Mobility assistive device | -.069 | .016 | -.175 | -4.421 | .000 | -.099 | -.038 | .931 | 1.074 |
|  | Educational level | .114 | .019 | .235 | 5.917 | .000 | .076 | .152 | .921 | 1.086 |
|  | Weekly Physical Activity | .002 | .000 | .255 | 6.595 | .000 | .001 | .002 | .975 | 1.026 |
| a. Dependent Variable: RSES score | | | | | | | | | | |
| b. Weighted Least Squares Regression - Weighted by weight RSES score | | | | | | | | | | |

***4-Without type of disability***

| **Coefficients^a,b^** | | | | | | | | | | |
| --- | --- | --- | --- | --- | --- | --- | --- | --- | --- | --- |
| Model | | Unstandardized Coefficients | | Standardized Coefficients | t | Sig. | 95.0% Confidence Interval for B | | Collinearity Statistics | |
|  |  | B | Std. Error | Beta |  |  | Lower Bound | Upper Bound | Tolerance | VIF |
| 1 | (Constant) | 2.595 | .139 |  | 18.628 | .000 | 2.322 | 2.869 |  |  |
|  | Gender | .016 | .045 | .016 | .357 | .721 | -.072 | .104 | .767 | 1.303 |
|  | Age (years) | .003 | .002 | .051 | 1.237 | .217 | -.002 | .007 | .872 | 1.147 |
|  | Mobility assistive device | -.065 | .016 | -.165 | -4.113 | .000 | -.096 | -.034 | .912 | 1.096 |
|  | Educational level | .114 | .020 | .234 | 5.830 | .000 | .075 | .152 | .912 | 1.096 |
|  | Weekly Physical Activity | .002 | .000 | .273 | 6.520 | .000 | .001 | .002 | .839 | 1.191 |
| a. Dependent Variable: RSES score | | | | | | | | | | |
| b. Weighted Least Squares Regression - Weighted by weight RSES score | | | | | | | | | | |

**Positive self-esteem**

***1- All demographic variables***

| Model | | Unstandardized Coefficients | | Standardized Coefficients | t | Sig. | 95.0% Confidence Interval for B | | Collinearity Statistics | |
| --- | --- | --- | --- | --- | --- | --- | --- | --- | --- | --- |
|  |  | B | Std. Error | Beta |  |  | Lower Bound | Upper Bound | Tolerance | VIF |
| 1 | (Constant) | 2.883 | .163 |  | 17.671 | .000 | 2.563 | 3.204 |  |  |
|  | Gender | -.015 | .051 | -.014 | -.306 | .759 | -.115 | .084 | .752 | 1.330 |
|  | Age (years) | -.003 | .002 | -.051 | -1.232 | .218 | -.008 | .002 | .877 | 1.141 |
|  | Type of physical disability | .029 | .013 | .087 | 2.196 | .028 | .003 | .055 | .951 | 1.052 |
|  | Mobility assistive device | -.034 | .018 | -.077 | -1.889 | .059 | -.070 | .001 | .891 | 1.122 |
|  | Educational level | .101 | .023 | .179 | 4.429 | .000 | .056 | .146 | .919 | 1.089 |
|  | Weekly Physical Activity | .002 | .000 | .280 | 6.493 | .000 | .001 | .002 | .803 | 1.245 |
| a. Dependent Variable: Positive feelings | | | | | | | | | | |
| b. Weighted Least Squares Regression - Weighted by Weight Positive feelings | | | | | | | | | | |

***2- Without age***

| Model | | Unstandardized Coefficients | | Standardized Coefficients | t | Sig. | 95.0% Confidence Interval for B | | Collinearity Statistics | |
| --- | --- | --- | --- | --- | --- | --- | --- | --- | --- | --- |
|  |  | B | Std. Error | Beta |  |  | Lower Bound | Upper Bound | Tolerance | VIF |
| 1 | (Constant) | 2.748 | .121 |  | 22.735 | .000 | 2.511 | 2.985 |  |  |
|  | Gender | -.003 | .050 | -.003 | -.057 | .954 | -.100 | .094 | .784 | 1.275 |
|  | Type of physical disability | .027 | .013 | .082 | 2.071 | .039 | .001 | .053 | .962 | 1.039 |
|  | Mobility assistive device | -.032 | .018 | -.072 | -1.759 | .079 | -.067 | .004 | .903 | 1.107 |
|  | Educational level | .107 | .022 | .189 | 4.796 | .000 | .063 | .151 | .962 | 1.040 |
|  | Weekly Physical Activity | .002 | .000 | .284 | 6.578 | .000 | .001 | .002 | .806 | 1.240 |
| a. Dependent Variable: Positive feelings | | | | | | | | | | |
| b. Weighted Least Squares Regression - Weighted by Weight Positive feelings | | | | | | | | | | |

***3- Without gender***

| Model | | Unstandardized Coefficients | | Standardized Coefficients | t | Sig. | 95.0% Confidence Interval for B | | Collinearity Statistics | |
| --- | --- | --- | --- | --- | --- | --- | --- | --- | --- | --- |
|  |  | B | Std. Error | Beta |  |  | Lower Bound | Upper Bound | Tolerance | VIF |
| 1 | (Constant) | 2.854 | .132 |  | 21.635 | .000 | 2.595 | 3.113 |  |  |
|  | Age (years) | -.003 | .002 | -.048 | -1.196 | .232 | -.007 | .002 | .914 | 1.094 |
|  | Type of physical disability | .029 | .013 | .087 | 2.189 | .029 | .003 | .055 | .951 | 1.051 |
|  | Mobility assistive device | -.035 | .018 | -.080 | -1.989 | .047 | -.070 | .000 | .927 | 1.079 |
|  | Educational level | .102 | .023 | .180 | 4.469 | .000 | .057 | .147 | .924 | 1.082 |
|  | Weekly Physical Activity | .002 | .000 | .286 | 7.285 | .000 | .001 | .002 | .971 | 1.029 |
| a. Dependent Variable: Positive feelings | | | | | | | | | | |
| b. Weighted Least Squares Regression - Weighted by Weight Positive feelings | | | | | | | | | | |

***4-Without type of disability***

| Model | | Unstandardized Coefficients | | Standardized Coefficients | t | Sig. | 95.0% Confidence Interval for B | | Collinearity Statistics | |
| --- | --- | --- | --- | --- | --- | --- | --- | --- | --- | --- |
|  |  | B | Std. Error | Beta |  |  | Lower Bound | Upper Bound | Tolerance | VIF |
| 1 | (Constant) | 2.983 | .157 |  | 18.976 | .000 | 2.674 | 3.292 |  |  |
|  | Gender | -.012 | .051 | -.011 | -.239 | .811 | -.112 | .088 | .753 | 1.329 |
|  | Age (years) | -.002 | .002 | -.041 | -.991 | .322 | -.007 | .002 | .888 | 1.127 |
|  | Mobility assistive device | -.029 | .018 | -.065 | -1.605 | .109 | -.064 | .006 | .907 | 1.102 |
|  | Educational level | .100 | .023 | .176 | 4.352 | .000 | .055 | .145 | .919 | 1.088 |
|  | Weekly Physical Activity | .002 | .000 | .294 | 6.869 | .000 | .001 | .003 | .821 | 1.218 |
| a. Dependent Variable: Positive feelings | | | | | | | | | | |
| b. Weighted Least Squares Regression - Weighted by Weight Positive feelings | | | | | | | | | | |

**Negative self-esteem**

***1- All demographic variables***

| Model | | Unstandardized Coefficients | | Standardized Coefficients | t | Sig. | 95.0% Confidence Interval for B | | Collinearity Statistics | |
| --- | --- | --- | --- | --- | --- | --- | --- | --- | --- | --- |
|  |  | B | Std. Error | Beta |  |  | Lower Bound | Upper Bound | Tolerance | VIF |
| 1 | (Constant) | 2.442 | .196 |  | 12.455 | .000 | 2.057 | 2.827 |  |  |
|  | Gender | .060 | .062 | .042 | .967 | .334 | -.061 | .181 | .776 | 1.289 |
|  | Age (years) | .004 | .003 | .061 | 1.470 | .142 | -.001 | .010 | .870 | 1.150 |
|  | Type of physical disability | .033 | .015 | .084 | 2.124 | .034 | .002 | .063 | .954 | 1.049 |
|  | Mobility assistive device | -.153 | .024 | -.263 | -6.517 | .000 | -.200 | -.107 | .914 | 1.094 |
|  | Educational level | .133 | .026 | .210 | 5.167 | .000 | .082 | .183 | .904 | 1.107 |
|  | Weekly Physical Activity | .002 | .000 | .204 | 4.753 | .000 | .001 | .003 | .810 | 1.235 |
| a. Dependent Variable: Negative feelings | | | | | | | | | | |
| b. Weighted Least Squares Regression - Weighted by Weight Negative feelings | | | | | | | | | | |

***2- Without age***

| Model | | Unstandardized Coefficients | | Standardized Coefficients | t | Sig. | 95.0% Confidence Interval for B | | Collinearity Statistics | |
| --- | --- | --- | --- | --- | --- | --- | --- | --- | --- | --- |
|  |  | B | Std. Error | Beta |  |  | Lower Bound | Upper Bound | Tolerance | VIF |
| 1 | (Constant) | 2.636 | .145 |  | 18.200 | .000 | 2.352 | 2.921 |  |  |
|  | Gender | .042 | .060 | .030 | .688 | .492 | -.077 | .160 | .808 | 1.238 |
|  | Type of physical disability | .036 | .015 | .092 | 2.350 | .019 | .006 | .066 | .973 | 1.028 |
|  | Mobility assistive device | -.158 | .023 | -.271 | -6.760 | .000 | -.204 | -.112 | .930 | 1.075 |
|  | Educational level | .125 | .025 | .197 | 4.961 | .000 | .075 | .174 | .949 | 1.054 |
|  | Weekly Physical Activity | .002 | .000 | .199 | 4.641 | .000 | .001 | .003 | .815 | 1.226 |
| a. Dependent Variable: Negative feelings | | | | | | | | | | |
| b. Weighted Least Squares Regression - Weighted by Weight Negative feelings | | | | | | | | | | |

***3- Without gender***

| Model | | Unstandardized Coefficients | | Standardized Coefficients | t | Sig. | 95.0% Confidence Interval for B | | Collinearity Statistics | |
| --- | --- | --- | --- | --- | --- | --- | --- | --- | --- | --- |
|  |  | B | Std. Error | Beta |  |  | Lower Bound | Upper Bound | Tolerance | VIF |
| 1 | (Constant) | 2.562 | .152 |  | 16.905 | .000 | 2.264 | 2.860 |  |  |
|  | Age (years) | .004 | .003 | .053 | 1.304 | .193 | -.002 | .009 | .906 | 1.104 |
|  | Type of physical disability | .033 | .015 | .085 | 2.162 | .031 | .003 | .064 | .955 | 1.047 |
|  | Mobility assistive device | -.150 | .023 | -.258 | -6.445 | .000 | -.196 | -.104 | .933 | 1.071 |
|  | Educational level | .130 | .026 | .206 | 5.095 | .000 | .080 | .181 | .913 | 1.095 |
|  | Weekly Physical Activity | .002 | .000 | .187 | 4.778 | .000 | .001 | .002 | .974 | 1.026 |
| a. Dependent Variable: Negative feelings | | | | | | | | | | |
| b. Weighted Least Squares Regression - Weighted by Weight Negative feelings | | | | | | | | | | |

***4-Without type of disability***

| Model | | Unstandardized Coefficients | | Standardized Coefficients | t | Sig. | 95.0% Confidence Interval for B | | Collinearity Statistics | |
| --- | --- | --- | --- | --- | --- | --- | --- | --- | --- | --- |
|  |  | B | Std. Error | Beta |  |  | Lower Bound | Upper Bound | Tolerance | VIF |
| 1 | (Constant) | 2.545 | .191 |  | 13.352 | .000 | 2.170 | 2.919 |  |  |
|  | Gender | .065 | .062 | .046 | 1.045 | .296 | -.057 | .186 | .777 | 1.287 |
|  | Age (years) | .005 | .003 | .073 | 1.779 | .076 | -.001 | .011 | .887 | 1.127 |
|  | Mobility assistive device | -.147 | .023 | -.253 | -6.289 | .000 | -.193 | -.101 | .927 | 1.078 |
|  | Educational level | .130 | .026 | .206 | 5.054 | .000 | .080 | .181 | .906 | 1.104 |
|  | Weekly Physical Activity | .002 | .000 | .215 | 5.034 | .000 | .001 | .003 | .822 | 1.217 |
| a. Dependent Variable: Negative feelings | | | | | | | | | | |
| b. Weighted Least Squares Regression - Weighted by Weight Negative feelings | | | | | | | | | | |
